# Quantitative modeling of lenticulostriate arteries on 7-T TOF-MRA for cerebral small vessel disease

**DOI:** 10.1186/s41747-024-00512-7

**Published:** 2024-11-05

**Authors:** Zhixin Li, Dongbiao Sun, Chen Ling, Li Bai, Jinyuan Zhang, Yue Wu, Yun Yuan, Zhaoxia Wang, Zhe Wang, Yan Zhuo, Rong Xue, Zihao Zhang

**Affiliations:** 1 State Key Laboratory of Brain and Cognitive Science, Institute of Biophysics, Chinese Academy of Sciences, 100101 Beijing, China; 2 Anhui Province Key Laboratory of Biomedical Imaging and Intelligent Processing, Institute of Artificial Intelligence, Hefei Comprehensive National Science Center, 230088 Hefei, China; 3 University of Chinese Academy of Sciences, 100049 Beijing, China; 4 Department of Neurology, Peking University First Hospital, Beijing, China; 5 Beijing Key Laboratory of Neurovascular Disease Discovery, Beijing, China; 6 Beijing Institute of Brain Disorders, Capital Medical University, Beijing, China

**Keywords:** Arteries, Brain, CADASIL, Magnetic resonance angiography, Neural networks (computer)

## Abstract

**Background:**

We developed a framework for segmenting and modeling lenticulostriate arteries (LSAs) on 7-T time-of-flight magnetic resonance angiography and tested its performance on cerebral autosomal dominant arteriopathy with subcortical infarcts and leukoencephalopathy (CADASIL) patients and controls.

**Methods:**

We prospectively included 29 CADASIL patients and 21 controls. The framework includes a small-patch convolutional neural network (SP-CNN) for fine segmentation, a random forest for modeling LSAs, and a screening model for removing wrong branches. The segmentation performance of our SP-CNN was compared to competitive networks. External validation with different resolution was performed on ten patients with aneurysms. Dice similarity coefficient (DSC) and Hausdorff distance (HD) between each network and manual segmentation were calculated. The modeling results of the centerlines, diameters, and lengths of LSAs were compared against manual labeling by four neurologists.

**Results:**

The SP-CNN achieved higher DSC (92.741 ± 2.789, mean ± standard deviation) and lower HD (0.610 ± 0.141 mm) in the segmentation of LSAs. It also outperformed competitive networks in the external validation (DSC 82.6 ± 5.5, HD 0.829 ± 0.143 mm). The framework *versus* manual difference was lower than the manual inter-observer difference for the vessel length of primary branches (median -0.040 mm, interquartile range -0.209 to 0.059 mm) and secondary branches (0.202 mm, 0.016–0.537 mm), as well as for the offset of centerlines of primary branches (0.071 mm, 0.065–0.078 mm) and secondary branches (0.072, 0.064–0.080 mm), with *p* < 0.001 for all comparisons.

**Conclusion:**

Our framework for LSAs modeling/quantification demonstrated high reliability and accuracy when compared to manual labeling.

**Trial registration:**

NCT05902039 (https://clinicaltrials.gov/study/NCT05902039?cond=NCT05902039).

**Relevance statement:**

The proposed automatic segmentation and modeling framework offers precise quantification of the morphological parameters of lenticulostriate arteries. This innovative technology streamlines diagnosis and research of cerebral small vessel disease, eliminating the burden of manual labeling, facilitating cohort studies and clinical diagnosis.

**Key Points:**

- The morphology of LSAs is important in the diagnosis of CSVD but difficult to quantify.
- The proposed algorithm achieved the performance equivalent to manual labeling by neurologists.
- Our method can provide standardized quantitative results, reducing radiologists’ workload in cohort studies.

**Graphical Abstract:**

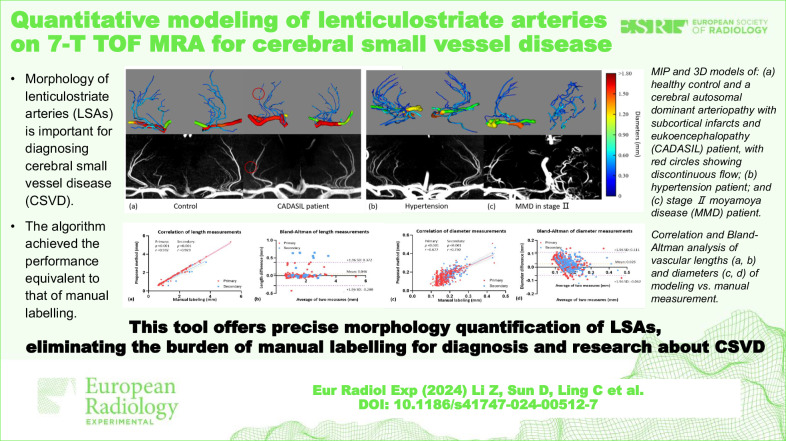

## Background

Cerebrovascular small vessel disease (CSVD) refers to abnormalities in small blood vessels that may lead to significant risk for cognitive impairment, dementia, stroke in the elderly, and neurological dysfunctions [1, 2]. Despite its clinical significance, detecting cerebral small vessels is difficult because of their small caliber and slow blood flow. Time-of-flight magnetic resonance angiography (TOF-MRA) at 7 T with improved contrast and higher spatial resolution is now a promising option for imaging cerebral small vessels, particularly the lenticulostriate arteries (LSAs) [3]. Studies using 7-T TOF-MRA revealed altered structure of LSAs’ in several conditions, including cerebral autosomal dominant arteriopathy with subcortical infarcts and leukoencephalopathy (CADASIL) [4], hypertension, and sporadic CSVD [5, 6]. However, the quantitative analysis of LSAs remains challenging because of the limited signal-to-noise ratio and pulsation artifacts in TOF-MRA images [7].

Deep learning has emerged as a viable solution to the challenge of segmenting and modeling LSAs, employing patch-based segmentation algorithms. Although classical image processing approaches [7] and machine learning [8] for LSAs segmentation and modeling are available, convolutional neural network (CNN) presents a particularly promising approach. Various CNN architectures, such as U-Net [9], multi-resolution CNN [10], multi-pathway CNN [11], and Res-Net [12], have been used for the segmentation of cerebral vessels. However, these networks commonly encounter a problem: segmentation on global images fails to adequately account for tubular structures of different diameters [8]. It is hypothesized that the intricate structure of CNN, characterized by multiple layers of convolution and pooling operations, might lead to the loss of information about finer structures.

To date, the literature lacks reports on the efficacy of deep learning methods for modeling cerebral small vessels that focus on the second and tertiary-level branches in 7-T TOF-MRA images [13]. Most existing studies have concentrated on modeling large vessels of the whole brain [10]. Moreover, these methods infrequently undergo comparison with physician-assessed results.

In this study, we developed a novel composite framework to segment and model LSAs on 7-T TOF-MRA images. The framework integrated a small-patch CNN (SP-CNN) for segmentation with a random forest model for subsequent vessel modeling. The segmentation performance was evaluated on images of CADASIL patients and various neurovascular diseases, and compared against existing neural networks. The modeling outcomes were compared with the manual labeling done by neurologists, revealing significant morphological differences in LSAs between CADASIL patients and the control group.

## Methods

Institutional review board approval of our prospective research was obtained from the Peking University First Hospital Biomedical Research Ethics Committee. Written informed consent was obtained from all the participants before enrollment in the study, which was registered under the clinical trials ID NCT05902039. Due to the privacy concerns regarding clinical data, the data are available from the corresponding author upon reasonable request for non-profit and scientific research purposes. The code of our study was accessible through the link: https://github.com/xingers/SP-CNN.

### Study participants and imaging protocol

We performed a prospective and observational study to investigate LSA in 29 CADASIL patients with gene diagnosis and 21 age- and sex-matched controls. Additionally, ten patients with intracranial aneurysms were included for external evaluation of the performance of SP-CNN. All participants underwent 7-T brain MRI scans at the Institute of Biophysics (Beijing, China) between 2016 and 2019.

The inclusion criteria for patients with CADASIL were as follows: (1) confirmed genetic diagnosis of CADASIL; (2) no history of acute ischemic/hemorrhagic events in the past 3 months; (3) the condition of the patient was stable and medication was not required for acute ischemic/hemorrhagic events; (4) age > 18 years. The World Federation of Neurology suggests that cerebral infarction stabilizes after 6 months, entering a chronic phase [14]. However, few CADASIL patients remain stable without bleeding or ischemic strokes in that time. Consequently, we revised the standard to require no severe strokes within 3 months, similar to a previous study [15]. The inclusion criteria for control participants were as follows: (1) no history of stroke or other major neurological disorders or severe systemic disorders; (2) age > 18 years. The exclusion criteria for both groups were as follows: (1) contraindication to MRI; and (2) inability to cooperate to complete the scan.

All MRI scans were performed on a 7-T MRI system (Siemens Healthineers, Erlangen, Germany) that was equipped with a 32-channel head coil (Nova Medical, Massachusetts, USA). Structural and vascular imaging were acquired for all participants. The three-dimensional (3D) TOF-MRA images were utilized to train and validate our algorithm, being acquired with the following technical parameters for both TOF-MRA in CADASIL patients and controls: spatial resolution 0.23 × 0.23 × 0.36 mm^3^; acquisition matrix 768 × 576 × 128; single slab; repetition time 15 ms; echo time 3.57 ms; flip angle 20°; bandwidth 15 1 Hz/Px, generalized autocalibrating partial parallel acquisition (GRAPPA) acceleration factor 2 with 60 reference lines; acquisition time 5:54 min:s. The TOF-MRA sequence in patients with intracranial aneurysms was acquired with the following technical parameters: spatial resolution 0.33 × 0.33 × 0.40 mm^3^; acquisition matrix 640 × 640 × 48; 6 slabs; repetition time 18 ms; echo time 4.96 ms; flip angle 24°; bandwidth 230 Hz/Px; GRAPPA acceleration factor 2 with 24 reference lines; acquisition time 9:16 min:s.

### Algorithm development

**Fig. 1:**
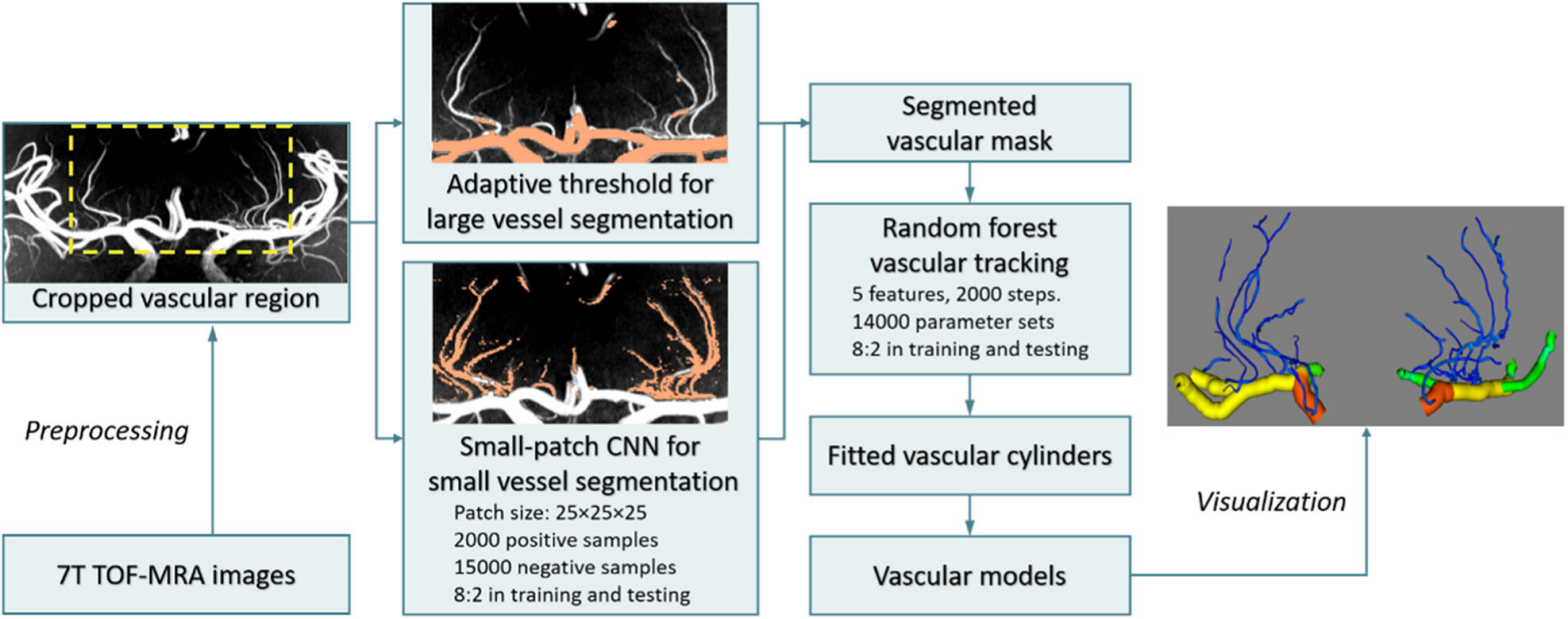
The architecture for the deep learning-based segmentation and tracking using random forest on 7-T time-of-flight magnetic resonance angiography

Our study designed a framework for segmentation and modeling of LSAs employing an SP-CNN coupled with a random forest approach (Fig. 1). The preprocessing involves N4 bias-field correction via SimpleITK (http://www.simpleitk.org) and denoising using the Pyramid Real Image Denoising Network [16], with subsequent rescaling of all images to an isotropic resolution of 0.10 mm for uniformity.

#### Segmentation phase

**Fig. 2:**
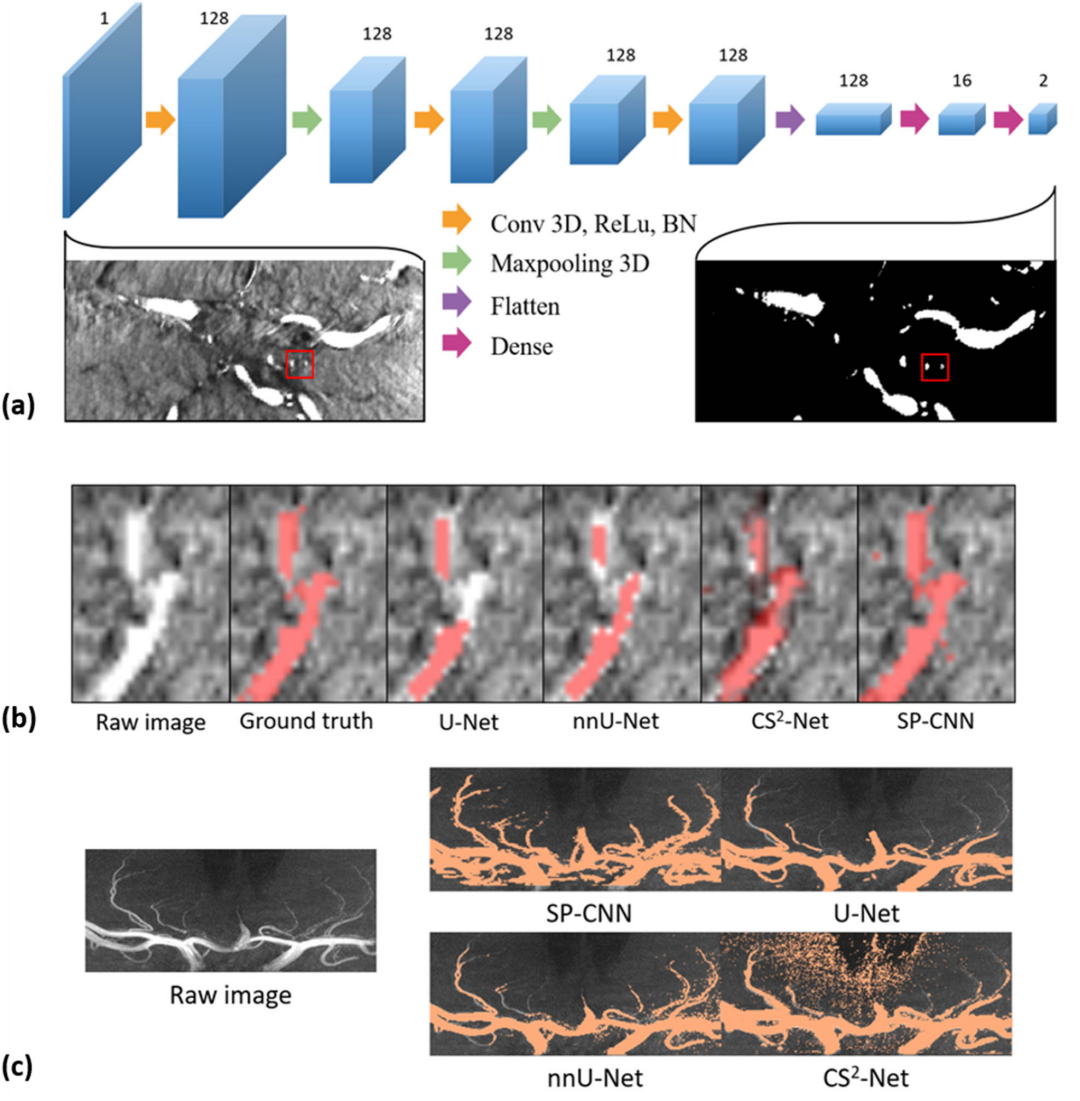
The proposed three-dimensional small-patch convolutional neural network (SP-CNN) for the segmentation of lenticulostriate arteries (LSA). **a** Network architecture; **b** comparison of segmentation results on a patch; **c** comparison of segmentation results on maximum intensity projection images

At the segmentation phase, SP-CNN was applied to the rescaled images. An illustrative depiction of the SP-CNN architecture is presented in Fig. 2a, with a detailed explanation of the theory provided in Supplementary Material A. To suitably capture the scale of LSAs, patch sizes were set at 25 × 25 × 25 voxels. A total of 17,000 training patches were derived from the images of seven control subjects. This dataset comprised 2,000 positive samples and 15,000 negative samples, distributed between the training and test sets at a ratio of 8:2. To ensure the model’s robustness to LSAs across various directions, 18 distinct local LSA trajectory orientations were included within the 2,000 positive samples, further detailed in Supplementary Material B.

Normalization of all input patches was conducted using the maximum and minimum values across the entire training dataset. The convolutional layers utilized a 3D kernel size of 3 with a stride of 2, employing the ReLU function for activation. Batch normalization layer followed each convolutional layer to mitigate the risk of gradient explosion or vanishing during training. The max pooling employed a 3D kernel size of 2. The network consisted of 128 feature maps in the initial layer, maintaining the number of feature maps through the down-sampling steps until the first dense layers. The output layer employed a sigmoid activation function for binary classification. The loss function was calculated as a sum of cross-entropy terms.

The SP-CNN was developed in the TensorFlow 2.1 framework (https://www.tensorflow.org) and trained on an Nvidia TITAN RTX GPU across 80 epochs, with a batch size of 25. Utilizing the Adam optimizer, we set an initial learning rate of 1e-4 and a minimum learning rate of 1e-8. A dynamic adjustment reduces the learning rate by a factor of 0.1 when the test loss fails to decrease after ten iterations. The training conducted with high-resolution patch images was stopped after 40 epochs to optimize model performance and prevent overfitting. The trends of the accuracy and the loss value in SP-CNN training were revealed in Supplementary Fig. S1.

When segmenting LSAs using SP-CNN, the signal intensities of large vessels (T_v_) and the background tissue (T_bg_) serve as the upper and lower boundaries, respectively, for the segmentation of cerebral small vessels. In the standardized images, these thresholds were set to 120 and 80.

#### Tracking stage

Tracking of LSAs began from the origins of the internal carotid arteries. The initial points of LSA and bifurcation points were detected using the spherical algorithm [17]. The small vascular cylinders were fitted using a random forest model on following features: (1) the proportion of the number of vascular voxels to the volume of the cylinder, (2) the variance of the distance from the center of the cylinder vertex to the edge of the vessel region, (3) the angle between the current vector and the previous vector, (4) the number of vessel voxels in a 5 × 5 × 5 area around the end of the cylinder, and (5) the eigenvalues of the Hessian matrix (σ = 1.5) in the 11 × 11 × 11 area centered on the cylinder endpoints in original images. For the training process, we employed 2,000 decision trees with 100 iterations and ensured randomness of the training samples in each iteration. A total of 14,000 target cylinder parameter sets were employed to train the random forest model (2,800 for training and 11,200 for testing), which were manually labeled from 50 vessels in two CADASIL patients and two control participants. To determine the vascular voxels within a cylinder, an energy loss function based on a minimal path approach was developed. The details can be found in Supplementary Material B.

#### Screening stage

A screening criterion was incorporated to reduce false-positive vascular branches. Branches were initially obtained after iterative tracking from initial vector and detecting bifurcation points with branch points as new initial vectors. However, a significant number of false positives existed in the vascular models. The reasons included repeated tracking on the same vessel, presence of pulsation artifacts (especially near the large vessels, such as middle cerebral artery and anterior cerebral artery), and scattered image noise leading to wrong connections. To solve these issues, a screening model was constructed, and details can be found in Supplementary Material C.

**Fig. 3:**
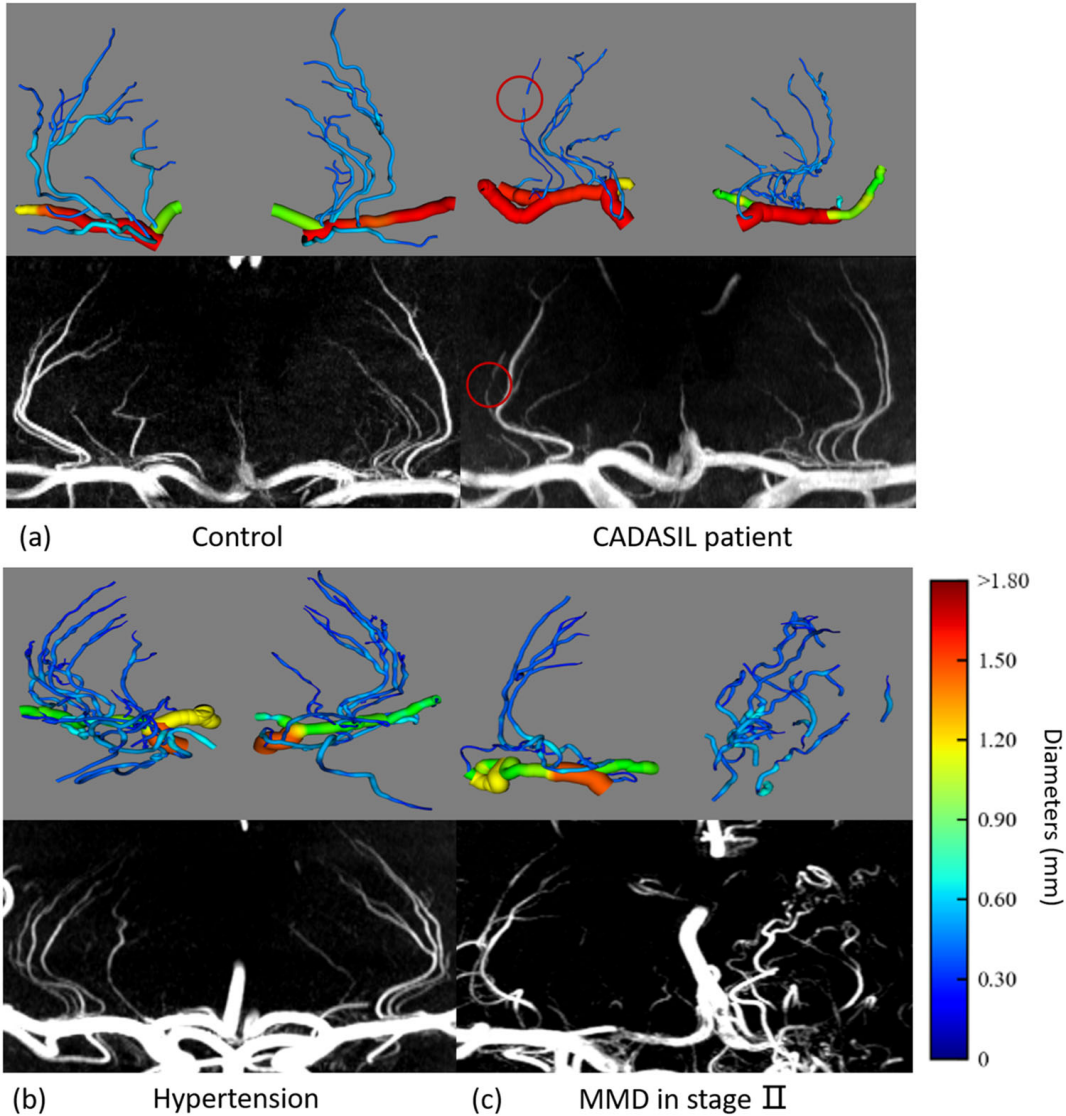
**a** Maximum intensity projection images and corresponding three-dimensional (3D) models of a control participant and a CADASIL patient. Regions with discontinuous flow signal are indicated by red circles. **b** Maximum intensity projection images and corresponding 3D models of a hypertension patient. **c** Maximum intensity projection images and corresponding 3D models of a MMD patient in stage II. CADASIL, cerebral autosomal dominant arteriopathy with subcortical infarcts and leukoencephalopathy; MMD, Moyamoya disease

After the screening step, branch centerlines were created by bifurcation judgments of interpolated third-order Bezier splines. The visualization was implemented using VTK package (VTK 9.1.0, Python 3.7). We applied information of centerlines and diameters to the generation of tubular structures, and the diameter of blood vessels was reflected in color, as shown in Fig. 3. Additionally, the geometric information, such as length, radius, quantity, and curvature, was derived from the above information. The 3D rotating views of the vasculatures and the overlaying views on raw images can be viewed via Supplementary Material Movies 1–4.

### Algorithm evaluation

The segmentation performance of the proposed SP-CNN was compared with U-Net, nnU-Net, and CS^2^-Net. The training parameters and details for these networks were described in Supplementary Table S1 and Supplementary Material D. For internal validation, we fed TOF-MRA images from five CADASIL patients and five controls into each of the above-mentioned neural networks for segmentation.

The TOF-MRA images of 29 CADASIL patients and 21 controls were manually labeled by four neurologists (F. Lin, Z. Li, D. Li, and Q. Kong) with varying levels of experience (3, 3, 4, and 6 years, respectively). They independently annotated the centerlines and diameter of LSA using the software Pair (https://www.aipair.com.cn/en/*)*. We calculated the offsets of centerline points and the differences in vascular diameter and length between the proposed method and the neurologists’ labeling. The error between the proposed algorithm and manual annotation was defined as inter-method variation, and the error between different readers was defined as inter-observer variation.

The robustness of the proposed algorithm was assessed across a variety of imaging parameters and diverse patient groups, in comparison with other networks. We conducted evaluations using a cohort of 62 patients with hypertension and hyperlipidemia (58.2 ± 10.558 years, 36 females (58.1%)), employing the following technical parameters for 7-T TOF-MRA: spatial resolution 0.36 × 0.36 × 0.4 mm^3^; acquisition matrix 448 × 560 × 169; repetition time 20 ms; echo time 3.57 ms; flip angle 24°; bandwidth 180 Hz/Px. For patients with subcortical infarction (*n* = 37), 7-T TOF-MRA was conducted with the following technical parameters: spatial resolution 0.31 × 0.31 × 0.3 mm^3^; acquisition matrix 600 × 656 × 168; repetition time 51 ms; echo time 5.61 ms; flip angle 20°; bandwidth 120 Hz/Px. In the case of patients with Moyamoya disease (MMD), the technical parameters were: spatial resolution 0.30 × 0.30 × 0.30 mm^3^; acquisition matrix 600 × 656 × 168; repetition time 16 ms; echo time 4.18 ms; flip angle 25°; bandwidth 180 Hz/Px. To compare with manual segmentation, ten maximum intensity projection (MIP) images for each of these groups of patients were labeled by two neurologists.

Additionally, to explore the tradeoff between segmentation quality and computation time, two neurologists, F. Lin and Z. Li (with 3 and 3 years of experience in brain MRI image segmentation, respectively), checked the segmentation results of our proposal method on the LSAs in the CADASIL patients and patients with infarction with voxel size from 0.10 mm to 0,20 mm. proposal method. When they tested the segmentation quality with different voxel sizes, they interpolated the origin TOF images to the different target voxel sizes as input for SP-CNN, as shown in Supplemental Fig. S3.

### Statistical analysis

The clinical characteristics of the two patient groups and controls were separately compared using independent sample *t*-tests. The segmentation performance of the networks was evaluated through internal and external validation. The false-positive (FP) rate was defined as the proportion of voxels incorrectly classified as vessels out of the total number of vessel voxels. The false negative (FN) rate referred to the proportion of voxels incorrectly classified as background out of the total number of background voxels. Two metrics, including Dice similarity coefficient (DSC) and Hausdorff distance (HD), were utilized between model prediction and manual segmentation, with the following definitions:

1 $${DSC}=\frac{2\times {TP}}{2\times {TP}+{FP}+{FN}}$$

2 $${HD}\left(P,G\right) =\,	\max \left(h\left(P,G\right),h\left(G,P\right)\right),{{{\rm{where}}}}\, h\left(P,G\right) \\ =\,	\max \left({\sum}_{p}^{P}\min \left({\sum}_{g}^{G}{{{\rm{||}}}}p-g{{{\rm{||}}}}\right)\right)$$

The manual segmentation was performed by a medical imaging student who was blinded to the clinical information of the data. To ensure the reliability of the results, we invited two neurologists, F. Lin and Z. Li, to examine the manually labeled results and received their approval. Paired *t*-tests were conducted between SP-CNN and the other networks. All statistical data were presented as mean ± standard deviation or as median(interquartile ranges), according to normal or non-normal distribution, respectively. We compared our framework to manual labeling, assessing differences in vascular diameters, lengths, and offsets of branch centerlines. Pearson correlation coefficients were calculated, and inter-method and inter-observer discrepancies were measured to evaluate credibility. A *p* < 0.05 indicated statistical significance.

To demonstrate the robustness of our methods, Bland-Altman analysis was utilized to assess the divergence between our method and other methods on two-dimensional MIPs with three different diseases, which is described in the section “Algorithm evaluation”.

## Results

### Participant characteristics

A total of 50 subjects were enrolled in our study, consisting of 21 controls and 29 CADASIL patients. Ten patients with aneurysms were used for external validation purposes. Table 1 presents the clinical characteristics of patients and control participants. The proportion of hypertension in CADASIL patients was significantly higher than that in the control group. Aneurysm patients were relatively older and had a higher proportion of hypertension than the control group. There were no significant differences in other clinical characteristics between the two patient groups and the control group.

**Table 1:** Clinical characteristics of the study participants

Characteristic	Controls (*n* = 21)	CADASIL patients (*n* = 29)	*p*-value	Aneurysm patients (*n* = 10)	*p*-value
Age, years (mean ± standard deviation)	47.5 ± 11.4	42.5 ± 11.4	0.132	59.0 ± 6.8	0.006
Sex, number of females (%)	25 (52.4%)	25 (51.7%)	0.964	8 (80.0%)	0.150
Body mass index (kg/m^2^)	23.4 ± 4.0	23.6 ± 3.0	0.809	23.8 ± 2.5	0.759
Hypertension (%)	0 (0.0%)	10 (34.5%)	0.002	2 (20.0%)	0.035
Hyperlipidemia (%)	1 (4.8%)	1 (3.4%)	0.820	2 (20.0%)	0.192
Hyperglycemia (%)	0 (0.0%)	0 (0.0%)	1.000	0 (0.0%)	1.000
Smoking history (%)	0 (0.0%)	0 (0.0%)	1.000	0 (0.0%)	1.000
Alcohol consumption (%)	0 (0.0%)	3 (10.3%)	0.139	1 (10.0%)	0.150

The two patient groups were each compared to the healthy control group using independent sample *t*-tests

*CADASIL* Cerebral autosomal dominant arteriopathy with subcortical infarcts and leukoencephalopathy

### Segmentation performance

Table 2 presents an overview of the segmentation performances of SP-CNN and several commonly used segmentation networks. In the internal validation, the developed SP-CNN delivered higher DSC values than that of 3D U-Net, 3D nnU-Net, and 3D CS^2^-Net. Additionally, the SP-CNN showed a lower HD compared to the candidate networks. The external validation results also indicated that SP-CNN outperformed all the candidate networks in DSC. Furthermore, the SP-CNN exhibited a lower HD than the candidate networks.

**Table 2:** False-positive rate, false negative rate, Dice similarity coefficient, and Hausdorff distance on small vessel segmentation using different models in both internal validation and external validation

	Model	FP (%)	FN (%)	DSC (%)	*p*-value	HD (mm)	*p*-value
Internal validation (*n* = 10)	U-Net	0.121 (0.075–0.135)	0.118 (0.084–0.129)	84.908 ± 1.731	< 0.001	0.867 ± 0.212	0.007
	nnU-Net	0.032 (0.023–0.035)	0.115 (0.058–0.136)	88.428 ± 1.174	< 0.001	0.712 ± 0.106	0.098
	CS^2^-Net	0.271 (0.223–0.286)	0.093 (0.058–0.105)	77.120 ± 1.816	< 0.001	0.932 ± 0.051	< 0.001
	SP-CNN	0.174 (0.083–0.205)	0.015 (0.009–0.018)	92.741 ± 2.789		0.610 ± 0.141	
External validation (*n* = 10)	U-Net	0.104 (0.074–0.114)	0.130 (0.068–0.152)	78.624 ± 7.931	0.091	1.151 ± 0.288	0.008
	nnU-Net	0.047 (0.037–0.050)	0.186 (0.109–0.211)	73.426 ± 6.619	0.005	1.250 ± 0.149	< 0.001
	CS^2^-Net	0.340 (0.286–0.358)	0.116 (0.077–0.129)	65.621 ± 4.678	< 0.001	1.285 ± 0.089	< 0.001
	SP-CNN	0.206 (0.109–0.237)	0.024 (0.011–0.029)	82.668 ± 5.540		0.829 ± 0.143	

Data in parentheses are interquartile ranges; *p*-values indicate the statistical significance when comparing U-Net, nnU-Net, and CS^2^-Net to the proposed SP-CNN

*DSC* Dice similarity coefficient, *FN* False negative, *FP* False-positive, *HD* Hausdorff distance, *SP-CNN* Small-patch convolutional neural network

Instances of vascular segmentation achieved by our proposed method and the models referenced in this paper are displayed in Fig. 2b. The results indicated that in vasculature with small calibers and bifurcations, our SP-CNN outperformed these networks.

### Modeling and measurements

The vasculature was reconstructed using splines and diameters obtained from tracking. Figure 3a provided 3D vascular models of a control participant and a CADIASIL patient, with color coding representing their diameter. Figure 3b provided 3D vascular models of a patient with hypertension. Figure 3c provided 3D vascular models of a MMD patient in stage II.

The discrepancies between our algorithm and manual labeling were summarized in Table 3, involving the offsets of the key points, and the differences in lengths and diameters. The key points distances of the centerlines were significantly smaller than the inter-observer distances of the key points marked by the four neurologists.

**Table 3:** Consistency analysis of the modeling results inter-methods (between the proposed method and manual labeling) and inter-observers, involving key point offset, length difference, and diameter differences

	Offset of key points (mm)		Difference of lengths (mm)		Difference of diameters (mm)	
	Primary	Secondary	Primary	Secondary	Primary	Secondary
Inter-method	0.071 (0.065–0.078)	0.072 (0.064–0.080)	-0.040 (-0.209 to 0.059)	0.202 (0.016–0.537)	0.094 (0.052–0.117)	0.090 (0.062–0.113)
Inter-observer	0.133 (0.087–0.182)	0.204 (0.137–0.264)	0.406 (0.262–0.632)	0.471 (0.333–0.763)	0.069 (0.060–0.100)	0.069 (0.062–0.084)
*p-*value	< 0.001	< 0.001	< 0.001	< 0.001	0.197	0.095

Data in parentheses are interquartile ranges. The *p*-value indicates the significant differences between the inter-method and inter-observer measurements

**Fig. 4:**
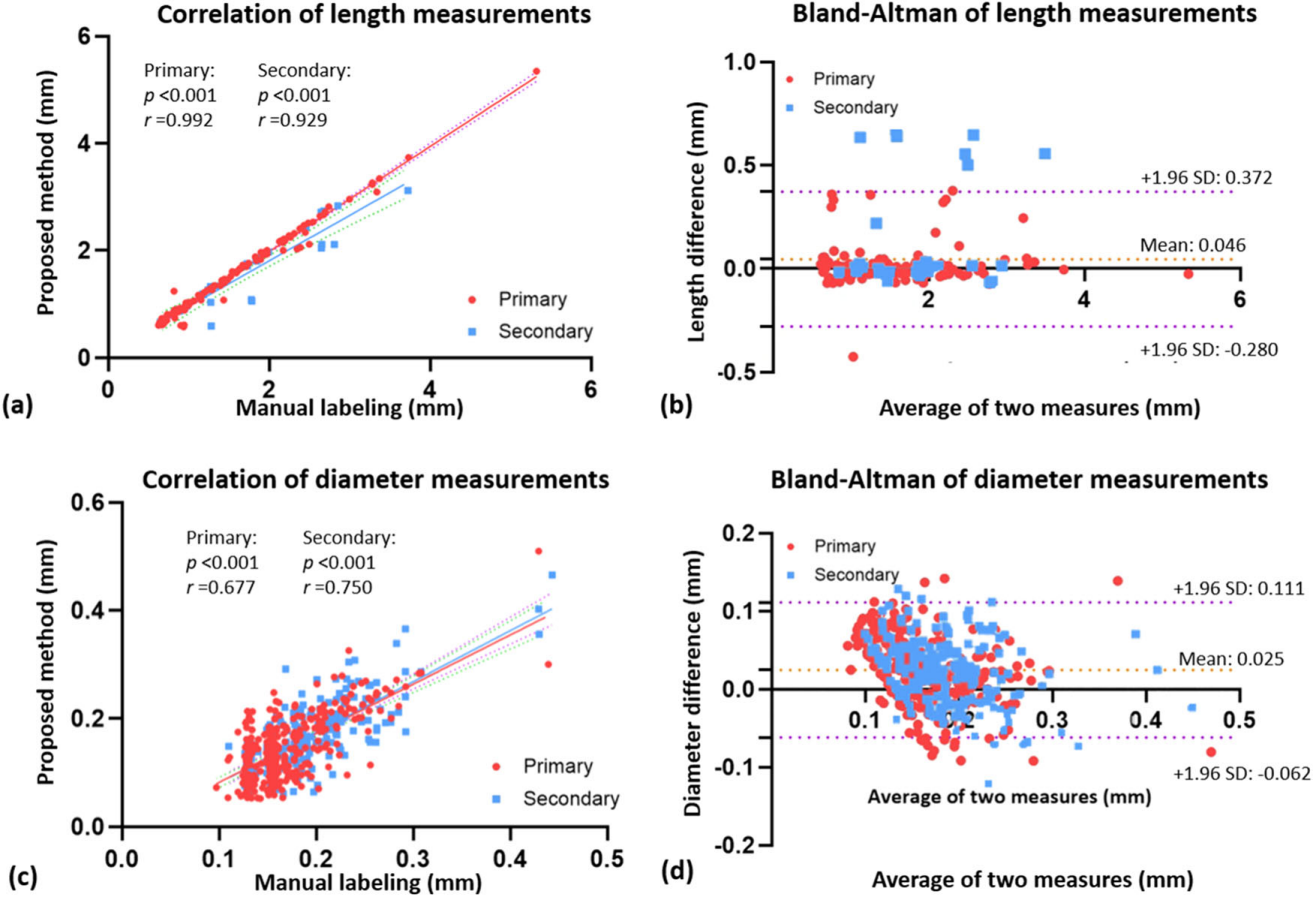
Correlation and Bland-Altman analysis of vascular lengths (**a**, **b**) and (**c**, **d**) diameters between the modeling results and manual measurement. SD, Standard deviation

The proposed method closely approximated the manual measurements of vascular length with strong correlation coefficients (Fig. 4a). The inter-method length differences on the primary and secondary branches were significantly smaller than the inter-observer differences. High correlation coefficients were also observed in the measurements of vascular diameters (Fig. 4c). The diameter differences between the automated and manual modeling were smaller than the voxel size (Fig. 4d).

In terms of computation time, our algorithm takes 1 h for the segmentation and 1 h for the tracking and modeling stage using a NVIDIA A800 GPU. In contrast, we have measured the time on five subjects that a skilled neurologist, D. Li, who has 4 years of experience and we mentioned above, required at least 3 h to obtain one structural data of all LSAs. The longest data consumption was 3 h and 30 min.

### Vascular features in diseased and healthy states

**Fig. 5:**
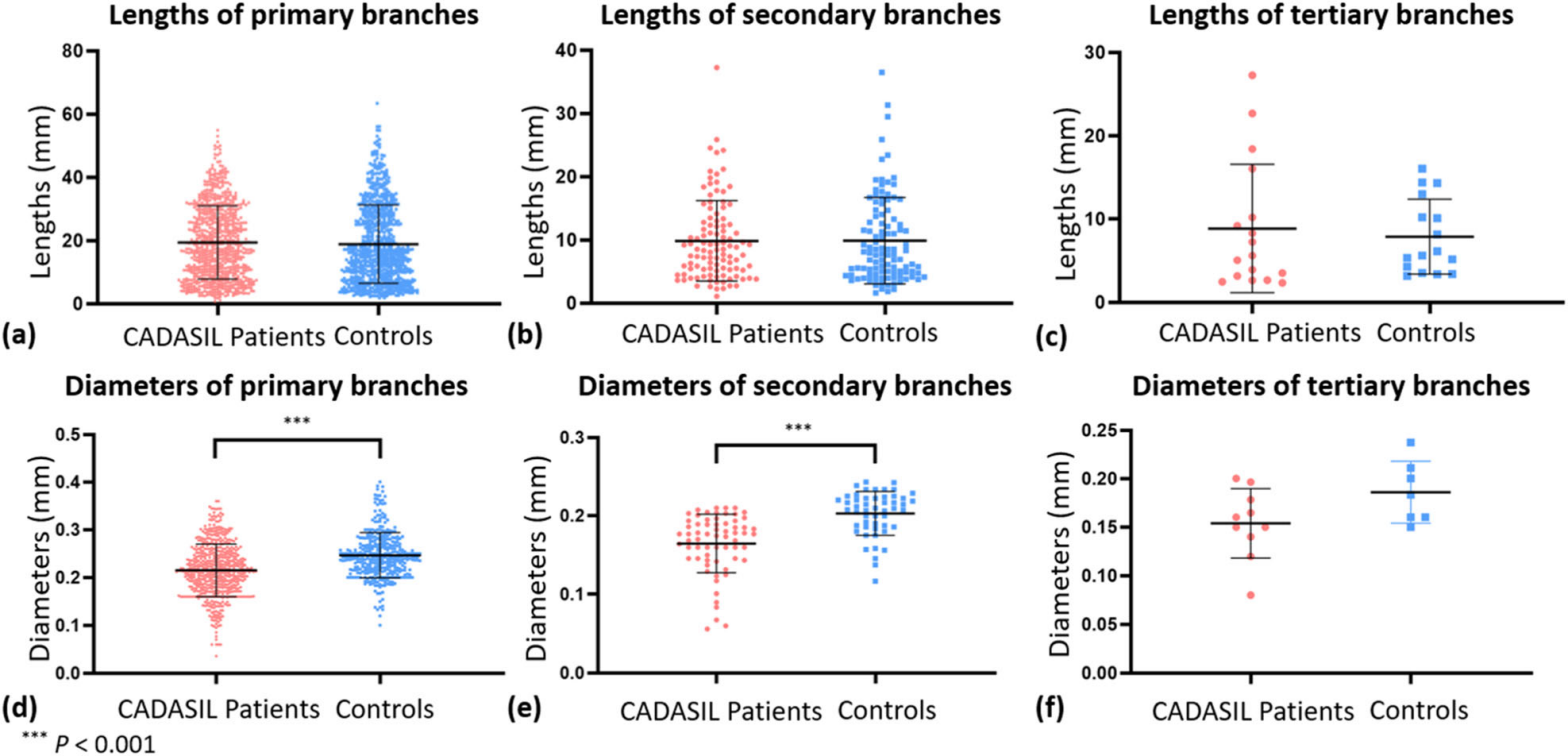
Lengths (**a**–**c**) and diameters (**d**–**f**) of different levels of LSA branches in CADASIL patients and healthy controls. CADASIL, Cerebral autosomal dominant arteriopathy with subcortical infarcts and leukoencephalopathy

Figure 5 presents the comparisons of lengths and diameters at three levels of LSA branches. CADASIL patients exhibited significantly smaller diameters in primary and secondary levels of LSA branches compared to controls. The statistical analysis revealed significantly smaller diameters in primary branches and secondary branches of LSA in CADASIL patients compared to controls. Additionally, there were no significant differences in the lengths of primary branches, secondary branches, and tertiary branches between the two groups. A receiver operating characteristic (ROC) analysis is presented in Supplementary Fig. S2, illustrating the significant differences in primary and secondary branches between controls and CADASIL patients.

### Robustness evaluation

**Fig. 6:**
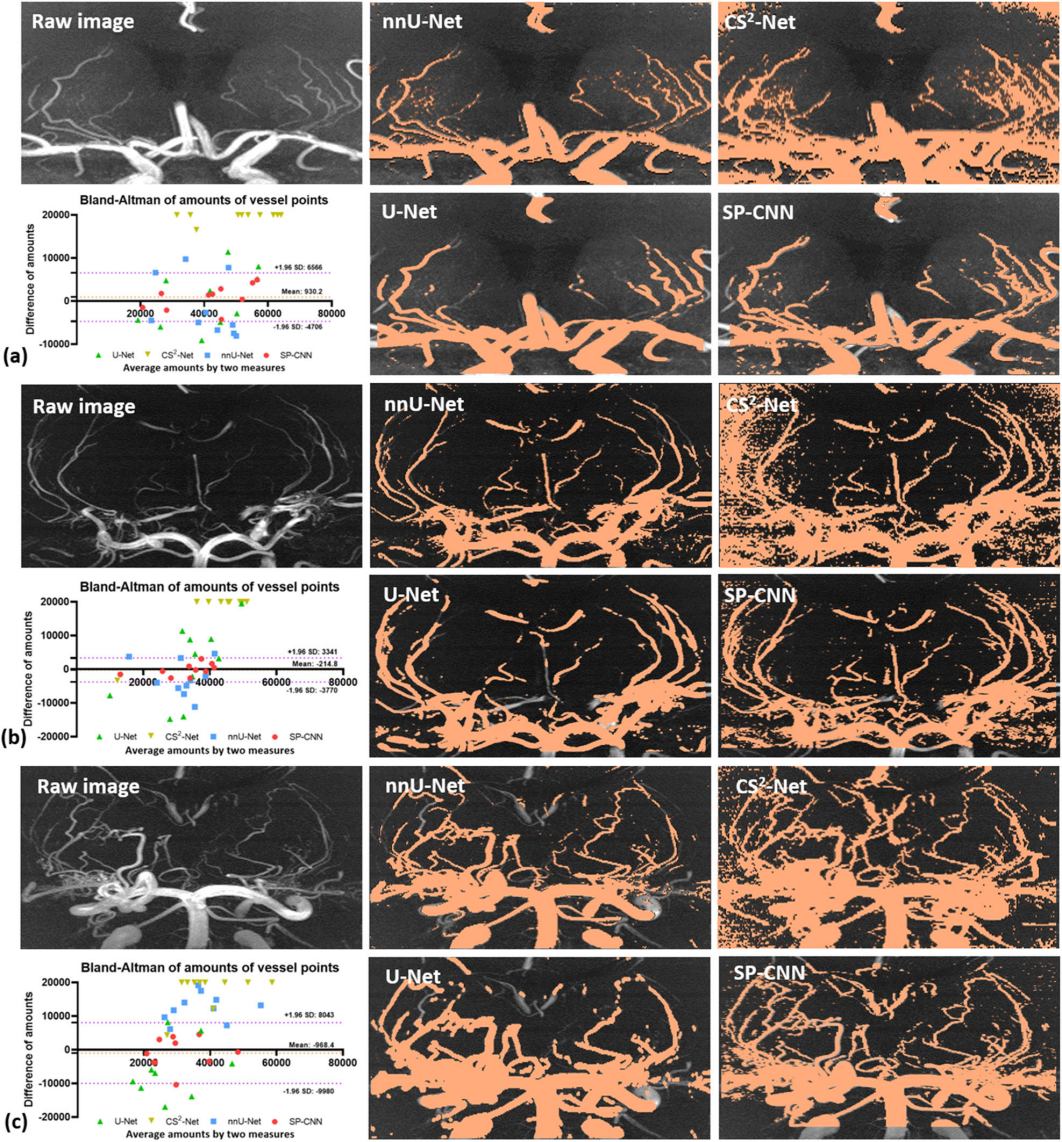
Comparison between our method and other approaches on 7-T time-of-flight magnetic resonance angiography acquired with various imaging parameters in diverse patient groups: (**a**) a patient with hypertension and hyperlipidemia; (**b**) a patient with infarction; and (**c**) a patient with Moyamoya disease. In these three Bland-Altman analysis results, the topmost points of CS^2^-Net represent the difference that exceeds the upper limit of the *y*-axis. The area between positive and negative 1.96 standard deviation in the Bland-Altman analysis represents the 95% confidence interval for our method. SP-CNN, Small-patch convolutional neural network

Figure 6 displays the comparative results of our proposed method against other networks on 7-T TOF-MRA images obtained with varying scanning parameters from three distinct patients: (a) a patient with hypertension and hyperlipidemia, (b) a patient with subcortical infarction, and (c) a patient with MMD. The Bland-Altman analysis of the MIP segmentations revealed the advantage of our SP-CNN compared to competitive networks among the three different diseases mentioned above. Supplementary Fig. S3 presented the time efficiency associated with using different interpolated voxel sizes in our proposal method.

## Discussion

In this study, we presented an accurate and robust framework for generating 3D models of LSA vasculature using 7-T TOF-MRA images and delineating their morphological characteristics. The framework was validated in patients with CADASIL and compared to manual labeling conducted by neurologists.

The results showed that the SP-CNN achieved higher DSC and lower HD in segmentation compared to other neural networks. Moreover, the framework’s ability to precisely quantify the centerline offsets, lengths, and diameters of LSA underscores its potential to significantly alleviate the clinical workload and facilitate future cohort studies in CSVD. The advent of ultra-high-field MRI has enhanced the visibility of minuscule anatomical structures, prioritizing the need for their accurate segmentation and quantification for both scientific research and clinical application [18, 19]. However, there is limited research on modeling methods of LSAs. Preceding studies have engaged in model-based segmentation and a 3D minimal path approach to visualize the primary and secondary LSA branches [7, 20], albeit without validation in patients diagnosed as CSVD. Our reconstruction demonstrates exceptional agreement with manual labeling, deviating by less than one voxel size, indicating the high credibility of our automated postprocessing in modeling cerebral small vessels.

Our results showed that the diameters of LSA branches obtained by our algorithm were significantly reduced in CADASIL patients. Supplementary Fig. S2 indicates that this measure can effectively distinguish CADASIL patients from healthy controls. Although previous literature holds reserved opinions about LSA narrowing in CADASIL patients [21, 22], some studies have shown that CADASIL leptomeningeal arteries exhibit advanced intimal hyperplasia and significant luminal narrowing [21, 23]. Additionally, hypertension may contribute to the narrowing of LSAs, as reported by Kang et al [24], which may play a confounding role in our results. The hypertension factor should be better controlled in future studies. Nonetheless, this result still indicated that our algorithm can sensitively detect structural abnormalities in LSAs [21–24].

The segmentation phase utilized SP-CNN, outperforming candidate networks in identifying intricate structures with minimized information loss. The 3D SP-CNN demonstrated superior segmentation performance across various datasets of vascular diseases, as demonstrated by the Bland-Altman analysis and the segmentation masks on MIPs in Fig. 6. Its effective segmentation of tertiary vessel branches is shown in Fig. 2c, while other networks only segmented primary branches and larger vessels [10, 25]. The reduction of effective receptive field in end-to-end network could explain their inferior performance in small vessel detection. Our method simplifies the segmentation of small vessels into a target detection problem by determining whether the center point of a small patch is part of a vessel, thereby achieving higher accuracy [26]. Notably, DSC alone may not fully represent the accuracy of our segmentation, as discrepancies in DSC could stem from mismatches in either a single large vessel or multiple small vessels [27, 28].

Another advantage of SP-CNN is its adaptability to images with varying resolution, unlike end-to-end networks that necessitate consistency in training and predicting structures, as shown in Fig. 3 and Fig. 6. In the tracking stage, five morphological features are introduced and tailored for precise cerebral small vessel tracking, effectively ensuring that the tracked splines remain confined to vascular structures, avoiding interference from other structures. Our approach surpasses traditional methods by leveraging information from the original image and the segmentation masks, thereby minimizing erroneous tracking of nonvascular artifacts resulting from arterial pulsation, head motion, and noise [29, 30].

While there are two drawbacks associated with the use of small patches, both have been well overcome in our study. First, large amounts of small patches led to increased computational costs. To address this issue, we set a grayscale interval of the small vessel area to pre-screen these voxels. The interval includes a higher threshold for representing the volume of large vessels and a lower threshold that was considered background. Second, this approach led to uniformly distributed false-positive signals due to inhomogeneous background signals. To solve this problem, we employed mean filtering for denoising and smoothing.

Meanwhile, there are still several limitations that cannot be overcome now in our study. First, the pulsation artifacts resulting from middle cerebral artery impeded the determination of the orifices of LSAs, which leads to hypothetical inference in tracking from large arteries to LSA stems. Second, since the algorithm generates large amounts of small patches from each image, it is relatively time-consuming. We have discussed several potential optimization strategies in Supplementary Material E, including adjustments to learning rates, batch sizes, and training epochs for the SP-CNN model. We also conducted preliminary tests on the impact of target resolution for interpolation on segmentation performance. The results in Fig. S3 indicated that moderately reducing the interpolation resolution can save computation time without losing small vessel details. Although all the neural networks we mentioned above for comparison got the same ground truth for training and testing, different models may be suitable for different datasets. Third, the manual segmentation and modeling were performed by a student who has 5 years of experience in medical imaging. In future studies on cerebral small vessels, higher-quality data annotations from more sophisticated radiologists are highly needed.

In conclusion, we showed that a framework for segmenting and modeling LSAs in 7-T TOF-MRA images, developed and tested on 29 patients with CADASIL and 21 controls, achieved a high DicDSC (92.741 ± 2.789) and a low HD (0.610 ± 0.141), outperforming other networks in both internal and external validation, and accurately quantified LSA lengths and diameters compared to manual labeling, with lower error than inter-observer variability in key point distances. This approach is expected to facilitate cohort studies on CSVD-related diseases, offering efficient and reliable quantification of LSA vasculatures.

## Supplementary information

**Additional file 1:** **Supplementary Table S1.** Hyper-parameter setups for the candidate networks used in the study. **Supplementary Figure S1.** Trends of the accuracy and the loss value in SP-CNN training. **Supplementary Figure S2.** The ROC curves showed the sensitivity and specificity of detecting CADASIL according to different diameter scales. **Supplementary Figure S3.** Segmentation results of SP-CNN by using different interpolated voxel sizes. The red circle indicates the blood vessels that were missed when the voxel size is reduced to isotropic 0.20 mm.

**Supplementary Movie 1:** The 3D vascular rotating view of a control participant.

**Supplementary Movie 2:** The overlaying view of 3D vasculatures on raw images of a control participant.

**Supplementary Movie 3:** The 3D vascular rotating view of a patient with CADASIL.

**Supplementary Movie 4:** The overlaying view of 3D vasculatures on raw images of a patient with CADASIL.

## Data Availability

Due to privacy concerns, individual deidentified participant data, images, and related documents will be shared after getting agreement from participant. The datasets generated or analyzed during the current study are available from the corresponding author upon reasonable request.

## Abbreviations

3D: Three-dimensional
CADASIL: Cerebral autosomal dominant arteriopathy with subcortical infarcts and leukoencephalopathy
CNN: Convolutional neural network
CSVD: Cerebral small vessel disease
DSC: Dice similarity coefficient
HD: Hausdorff distance
LSAs: Lenticulostriate arteries
MIP: Maximum intensity projection
MMD: Moyamoya disease
SP-CNN: Small-patch convolutional neural network
TOF-MRA: Time-of-flight magnetic resonance angiography

## Code availability

The code of our study is accessible through the link: https://github.com/xingers/SP-CNN.
