# Supplementary material for: Quantitative modeling of lenticulostriate arteries on 7-T TOF-MRA for cerebral small vessel disease
Source: Eur Radiol Exp. 2024 Nov 5;8:126. doi: 10.1186/s41747-024-00512-7 (PMC11538103; doi:10.1186/s41747-024-00512-7)
Supplement: Supplementary file 1 — Additional file 1: Supplementary Table S1. Hyper-parameter setups for the candidate networks used in the study. Supplementary Figure S1. Trends of the accuracy and the loss value in SP-CNN training. Supplementary Figure S2. The ROC curves showed the sensitivity and specificity of detecting CADASIL according to different diameter scales. Supplementary Figure S3. Segmentation results of SP-CNN by using different interpolated voxel sizes. The red circle indicates the blood vessels that were missed when the voxel size is reduced to isotropic 0.20 mm. [file 41747_2024_512_MOESM1_ESM.docx]

**Quantitative modeling of lenticulostriate arteries on 7-T TOF MRA for cerebral small vessel disease**

**ELECTRONIC SUPPLEMENTARY MATERIAL**

**Supplemental Figures and Tables:**

| **Network** | **Hyper-parameters** |
| --- | --- |
| **3D U-Net** | Batch size:64, Learning rate: 1e-5, Patch size: 64×64×64 |
| **3D CS^2^-Net** | Learning rate: 1e-5, Patch size: 64×64×64 |
| **nnU-Net** | Model: 3D full-resolution |

Table S1. Hyper-parameter setups for the candidate networks used in the study.


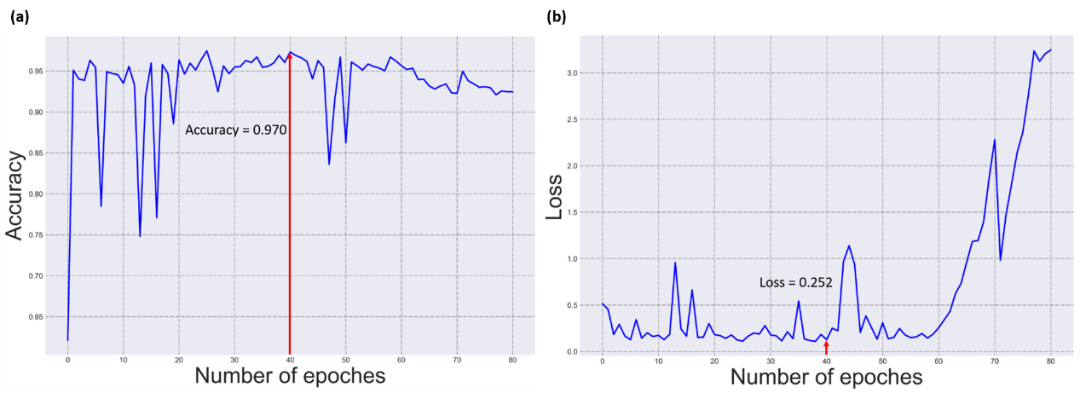


**Figure S1**. Trends of the accuracy and the loss value in SP-CNN training.


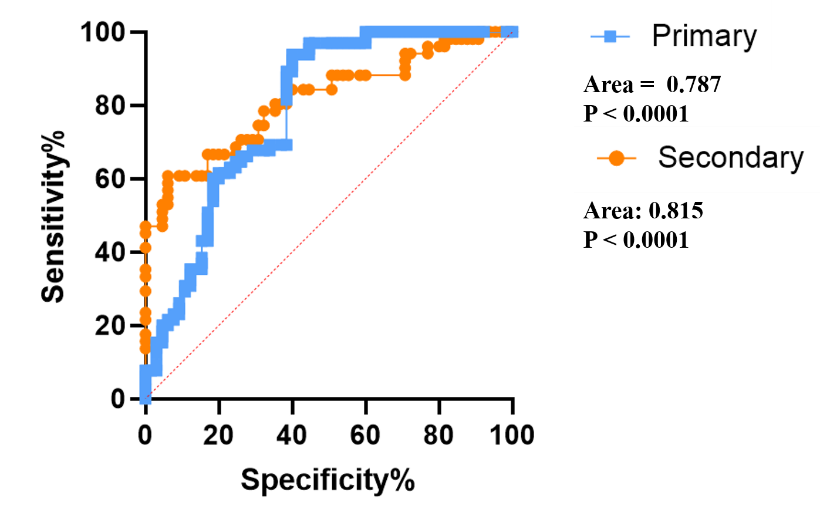


**Figure S2**. The ROC curves showed the sensitivity and specificity of detecting CADASIL according to different diameter scales.


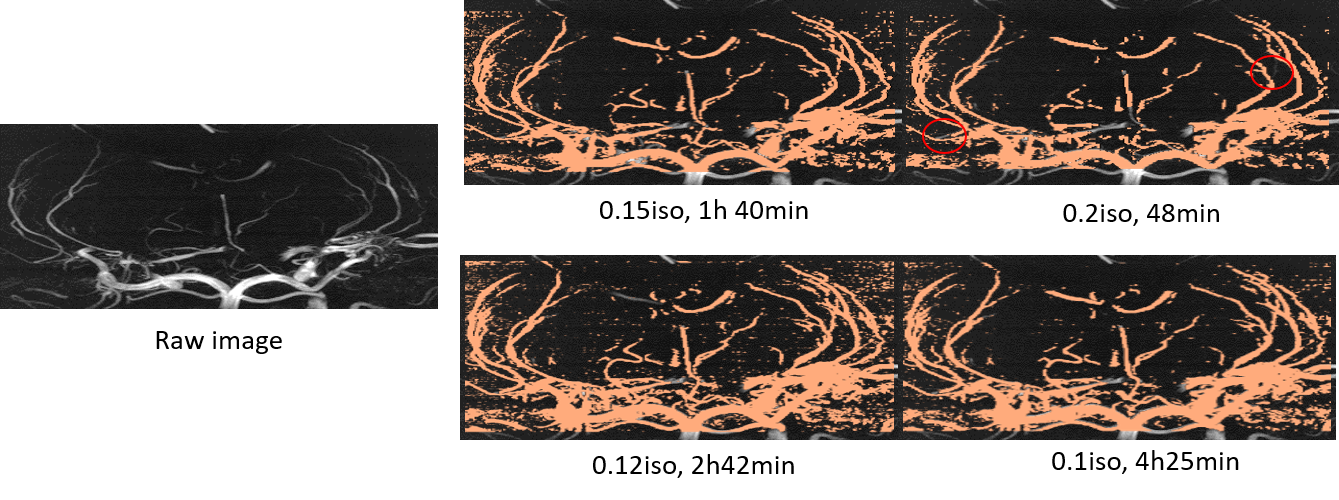


**Figure S3**. Segmentation results of SP-CNN by using different interpolated voxel sizes. The red circle indicates the blood vessels that were missed when the voxel size is reduced to isotropic 0.20 mm.

**Movie legends**

Movie 1. The 3D vascular rotating view of a control participant.

Movie 2. The overlaying view of 3D vasculatures on raw images of a control participant.

Movie 3. The 3D vascular rotating view of a patient with CADASIL.

Movie 4. The overlaying view of 3D vasculatures on raw images of a patient with CADASIL.

1. **The theory of SP-CNN**

As Shit[1] have mentioned that training on a globally averaged loss induces a strong bias towards the volumetric segmentation of large vessels, we analyzed the question as follow. In a 3D volume, 3D convolution can be expressed as the following formula:

$F_{m+1}(i,j,k)=\sum_{w} \sum_{h} \sum_{d} k\left( w,h,d \right)F_{m}\left( ⅈ+w,j+h,k+d \right)$ (1)

where *w, h, d* was the size of the convolution kernel and *i, j, k* was the position of the 3D image. We assumed that I_vessel_ > I_background_, and the average of I_L_ ≥ average I_S_, with I representing the signal intensity in the TOF-MRA image. L and S refer to large and small blood vessels, respectively. Obviously, the number of points from large vessels per unit volume N_L_ was greater than that from small vessels N_S_. Based on (1) and the above conditions, we had (2):

$\Sigma F_{s,m}<\Sigma F_{L,m}$ (2)

where *F* represents the feature map, and *m* represents the number of layers. When any of the *w, h, d* > 1, Eq. 3 was established:

$\Sigma F_{L,m}- \Sigma F_{s,m}<\Sigma F_{L,m+1}- \Sigma F_{s,m+1}$(3)

The growth brought about by convolution was nonlinear. As the convolution kernel size was greater than 1×1×1, even if the signal intensity of large vessels was same to small vessels, the growth of large vessels was still greater than that of small vessels on the feature map. When m > 0, we obtained (4) as follows:

${mean(F}_{L,m})> {mean(F}_{s,m})$ (4)

From (3) and (4), (5) can be derived:

${mean(F}_{L,m})/{mean(F}_{s,m})< {mean(F}_{L,m+1}){mean(F}_{s,m+1})$(5)

which means that in computer vision, the feature of small blood vessels would be closer to the background when *m* increased. However, if a patch that only contained LSAs was input to the network, the vessel and background can be clearly distinguished. To achieve higher accuracy in an easier manner [2], only center point will be predicted so that this problem was transformed to target detection instead of segmenting each voxel in the patch. In addition, due to the limited available dataset, noise information in the positive samples should be suppressed, otherwise the network may be misled in learning. The diameters of LSAs were usually less than 0.5mm. When the patch size is 5 times larger than the diameters in each direction, the second-order partial derivative can distinguish tubular structures, so we use patches with a scale of 2.5mm.

**B. Training in Tracking**

The small vascular cylinders were fitted using a random forest model on following features: 1) the proportion of the number of vascular voxels to the volume of the cylinder, 2) the variance of the distance from the center of the cylinder vertex to the edge of the vessel region, 3) the angle between the current vector and the previous vector, 4) the number of vessel voxels in a 5×5×5 area around the end of the cylinder, and 5) the eigenvalues of the Hessian matrix (σ = 1.5) in the 11×11×11 area centered on the cylinder endpoints in original images.

For the training process, we employed 2000 decision trees with 100 iterations and ensured randomness of the training samples in each iteration. A total of 14000 target cylinder parameter sets were employed to train the random forest model (2800 for training and 11200 for testing), which were manually labeled from 50 vessels in two CADASIL patients and two control participants.

To determine the vascular voxels within a cylinder, an energy loss function based on a minimal path approach was developed. The function used the intensity of the voxel *I*, the distance to the end point *D*, and the arccosine of the angle between the potential vector and the previous vector *A* as components of energy loss *E*_step_ at each step. The formula was as follows:

$E_{step}=\alpha I+\beta D+\delta A$ (6)

where *α, β, δ* were the weights of *I*, *D*, *A*. The total energy loss along the entire path was calculated as:

$E_{total}=\sum_{0}^{m} E_{step}$ (7)

where *m* was the number of points in the path. In each step of the tracking process, if selecting a cylinder vertex resulted in the energy loss (E*_total_*) surpassing the threshold, the process was prohibited. The threshold was obtained by calculating the minimum loss from 1000 disconnected samples and confirmed by two neurologists.

The bifurcation judgments were conducted on the centerlines of branches interpolated using a third-order Bezier spline. Bifurcation points were detected using the spherical algorithm [3, 4]. A vector was created from the bifurcation point on the origin vessel to the detected branch points, initiating a tracking process of the new branch.

**C. Screening Model and Visualization**

A screening model was constructed so that the following criteria must be satisfied: (1) The proportion of points on the current spline that aligned with existing vessel trunks was less than 30%; (2) The absolute value of the derivative of the diameter along the spline was less than 0.3 mm/vector;(3) The maximum curvature of the spline was less than 90°; (4) More than 70% of the centerline points had a variance of the three eigenvalues of the Hessian matrix (18x18x18, *σ* = 1.5) greater than 20; and (5) The energy loss function between two points of the initial vector was less than 30. Vessels that met these conditions were retained.

After screening step, branch centerlines were created by bifurcation judgments of interpolated third-order Bezier splines. The visualization was implemented using VTK package (VTK = 9.1.0, Python = 3.7). We applied information of centerlines and diameters to the generation of tubular structures, and the diameter of vessels was reflected in color.

**D. Setting of neural networks**

To assess the segmentation performance of SP-CNN for LSAs, the U-Net, nnU-Net, and CS^2^-NET were utilized as comparisons. Minibatch stochastic gradient descent was used to optimize the binary cross-entropy loss function for all the networks. The weights of the SP-CNN were optimized using the Adam optimizer with an exponentially decreasing learning rate of 1e^-4^ after initial warm-up steps [5]. All networks were trained from scratch using mini-batches of 64×64×64 if the patch size could be set. For nnU-Net, 3D full-resolution and 5-fold cross-validation were implemented.

**E. Training and Testing with SP-CNN**

The SP-CNN were implemented in TensorFlow framework 2.1 *(*<https://www.tensorflow.org>), and trained using an Nvidia TITAN RTX GPU for 80 epochs on each train. The batch-size of this model was 25. The Adam optimizer with an initial learning rate is set to 1e-4, the minimum learning rate is 1e-8, while we set a learning rate reducing function in call backs that the learning rate will be multiply 0.1 when the loss in testing no longer decreases by 10 times. The training of SP-CNN was performed using high-resolution patch images and stopped after 40 epochs, with detailed results shown in Figure S1. The training process consumes approximately 4 hours for the size of our training dataset. When using our SP-CNN to make predictions on images, the time consumed varies with the size of the images. For the image size of CADASIL patients that mentioned in the main-body, the time required is approximately 6 hours.

**References**

[1] Shit S, Paetzold JC, Sekuboyina A, et al (2021) clDice-a novel topology-preserving loss function for tubular structure segmentation, Proceedings of the IEEE/CVF Conference on Computer Vision and Pattern Recognition 16560–16569. https://doi.org/10.1109/cvpr46437.2021.01629

[2] Srinivasan G, Shobha G (2007) Segmentation techniques for target recognition, Int J Comput Commun 1:313–333.

[3] Arimura H, Li Q, Korogi Y, et al. (2004) Automated computerized scheme for detection of unruptured intracranial aneurysms in three-dimensional magnetic resonance angiography1, Acad Radiol 11: 1093-1104. <https://doi.org/10.1016/j.acra.2004.07.011>.

[4] Kumar RP, Albregtsen F, Reimers M, et al. (2015) Three-dimensional blood vessel segmentation and centerline extraction based on two-dimensional cross-section analysis, Ann Biomed Eng 43: 1223-1234.

[5] Kingma DP, Ba J (2014) Adam: A method for stochastic optimization, arXiv preprint arXiv:1412.6980.
